# A positive feedback-based gene circuit to increase the production of a membrane protein

**DOI:** 10.1186/1754-1611-4-6

**Published:** 2010-05-25

**Authors:** Karan Bansal, Ke Yang, Goutam J Nistala, Robert B Gennis, Kaustubh D Bhalerao

**Affiliations:** 1 Dow AgroSciences, Indianapolis, IN, USA; 2 Department of Biochemistry, University of Illinois at Urbana Champaign, USA; 3 Department of Agricultural and Biological Engineering, University of Illinois at Urbana Champaign, 1304 W. Pennsylvania Ave, Urbana, IL 61801 USA

## Abstract

**Background:**

Membrane proteins are an important class of proteins, playing a key role in many biological processes, and are a promising target in pharmaceutical development. However, membrane proteins are often difficult to produce in large quantities for the purpose of crystallographic or biochemical analyses.

**Results:**

In this paper, we demonstrate that synthetic gene circuits designed specifically to overexpress certain genes can be applied to manipulate the expression kinetics of a model membrane protein, cytochrome *bd *quinol oxidase in *E. coli*, resulting in increased expression rates. The synthetic circuit involved is an engineered, autoinducer-independent variant of the *lux *operon activator LuxR from *V. fischeri *in an autoregulatory, positive feedback configuration.

**Conclusions:**

Our proof-of-concept experiments indicate a statistically significant increase in the rate of production of the *bd *oxidase membrane protein. Synthetic gene networks provide a feasible solution for the problem of membrane protein production.

## Background

One of the goals of the emerging discipline of synthetic biology is to make it easy to 'reprogram' the behavior of living organisms by transforming them with novel, rationally engineered, synthetic gene networks. Engineered gene networks have numerous applications in diverse biotechnological endeavors such as metabolic engineering [1,2], single cell biosensors [3,4] as well as cell-based computers [5,6]. Precise regulation of gene expression either through exogenous control or through endogenous autoregulation is often critical for the development of synthetic biology applications. Several strategies have been developed for gene regulation including the development of libraries with modified promoter sequences [7], development of gene expression toggle switches controlled by exogenous inputs [8], gene expression pulse generators [9] and oscillators [10], and even post-transcriptional regulatory systems [11]. There are numerous reviews detailing the engineering principles behind the development of synthetic gene circuits, recent advances, and potential applications in the future [12-15].

Autoregulation by way of positive or negative feedback is commonly used as a motif in gene regulatory circuits. Depending on the specific circuit topology, feedback often leads to improved performance of the gene regulatory circuits. Specifically, a positive feedback within the regulatory circuit can lead to increased steady state level (amplification) of the gene expression, faster response kinetics, and increased sensitivity to exogenous inducers or autoinducers [16-19]. These properties are particularly suitable for applications such as protein production, where the benefits of increased production rates can directly translate into reduced resource costs and increased profits.

While gene circuits are routinely used to express soluble proteins such as green fluorescent protein (GFP) in model circuits, they have not been applied for the production of membrane proteins. The structure and biochemistry of membrane proteins are extremely important to study. About 20% of all genomes sequenced so far appear to encode membrane proteins [20]. Additionally, their key role in the etiology of many diseases make them targets for therapeutic intervention. 60-70% of all drug targets are membrane proteins, such as the G-protein coupled receptor family [21,22].

Membrane proteins are very difficult to produce [22]. They are normally expressed at very low levels in nature. It is possible to express them at higher levels using strong promoters such as the hybrid *tac *and *trc *promoters, or high-activity polymerases such as the T7 RNA polymerase, however there are some inherent drawbacks with these methods such as leaky expression or promoter toxicity. Additionally, heterologous expression of membrane proteins can cause aggregation of the product within the cells. These inclusion bodies have to be separated and refolded in order to reconstitute their native structure. This is often an inefficient and laborious process. We note that there are recent developments in the field of membrane protein expression, such as the development of tunable T7 RNA polymerase-based systems (the so called "Walker strains", C41(DE3) and C43(DE3)) that are not significantly affected by T7 RNAP toxicity [23]. Positive feedback-based synthetic gene circuits offer an alternative to the gene expression techniques discussed above. In this study, we explored the possibility of increasing the expression of the model membrane protein, cytochrome *bd *quinol oxidase (also referred to as *bd *oxidase), a terminal oxidase from *E. coli*. Normally, *bd *oxidase is expressed under microaerobic conditions since it has a high affinity to oxygen and serves as an oxygen scavenger in low oxygen conditions [24,25]. Further, the biochemistry of *bd *oxidase is well-characterized, and methods to measure the gene product levels spectrophotometrically are well established [26]. These features make *bd *oxidase a good test case to explore the positive feedback-based gene expression system.

## Materials and methods

### Design of the synthetic gene network

**Figure 1:**
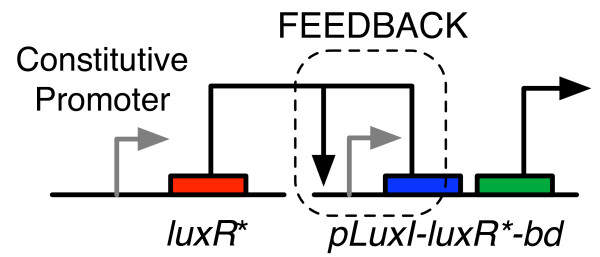
**A schematic showing the positive feedback-based gene expression system**.

A positive feedback circuit was built using elements from the quorum sensing system in *Vibrio fischeri *and the *bd *oxidase gene as shown in Figure 1. LuxR is a transcriptional activator from the *V. fischeri *quorum sensing system that binds to its cognate promoter *p*_*lux *_and activates expression of genes under its control. The wild type LuxR is inactive when produced. Acyl homoserine lactone (AHL), produced by another gene, *luxI*, is an autoinducer that binds LuxR and increases its activity. There are other similar positive feedback circuits based on the wild type LuxR, which rely on the action of AHL, either exogenously supplied, or produced by the action of *luxI*, for their expression system to operate correctly.

In our circuit, we specifically desired a regulatory protein that was not reliant on any external co-factors, and operated independent of the cell density in the culture. Fortunately, the structure-activity relationship of LuxR is well-known. Previous studies have determined that an Ala221→Val mutant of LuxR (denoted LuxR*) activates the *p*_*lux*_promoter even in the absence of AHL [19,27].

The gene network design consisted of two plasmids. The first plasmid encoded a copy of the luxR* gene under a constitutive promoter. The second plasmid encoded a copy of the *bd *oxidase gene, along with another copy of the luxR* gene in a bi-cistronic configuration. The two-plasmid system was chosen to ensure modularity in design and to enable the facile construction of experimental controls to validate the positive feedback.

### Selection of the host strain

*E. coli *strain *ML15A *is a *bd *oxidase knockout derivative of the "Walker strain" *C43 (DE3) *(*F^-^ompT hsdS*_*B *_()*gal dcm cyd^-^*), and was used as the host strain for expressing the wild-type *bd *oxidase from a plasmid. It has a tendency to overproduce membrane proteins [23]. All cloning work was performed by electroporation using electrocompetent *ML15A *cells. The *C43 (DE3) *wild type control does not have any plasmids, and has a chromosomal *bd *oxidase gene, and was used as a control for observing native expression of *bd *oxidase.

### Plasmid construction

Plasmids pGN3, pKB1 and pKB2 were based on the PRO™Tet6xHN bacterial expression system (Clontech Laboratories, Mountain View, CA). The PRO™Tet6xHN plasmid formed the basis of the plasmids constructed for this experiment, which are described below.

#### Constitutive plasmid

The ColE1 origin from PRO™Tet6xHN was replaced by p15A between the restriction sites XbaI and SacI as described by Lutz and Bujard [28]. The chloramphenicol resistance marker was replaced by the ampicillin resistance marker (Ap^*r*^) from pZE21 between the unique restriction enzyme sites XhoI and SacI yielding pPROTetE-Kan-p15A.

**Figure 2:**
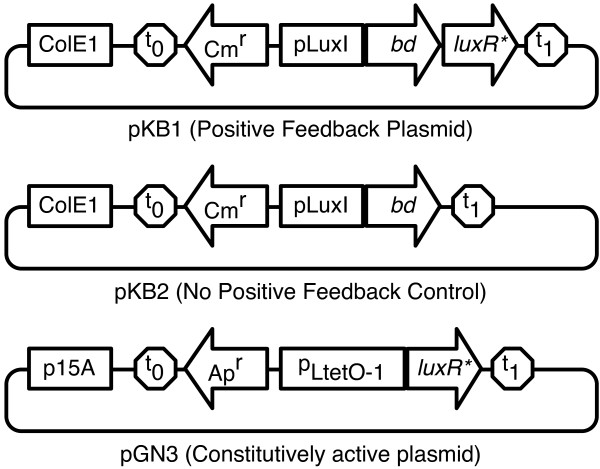
**Schematics showing the different circuits tested**. Plasmids pKB1(PF plasmid) and pKB2 (NPF control) are identical except for the absence of the luxR* element responsible for positive feedback on the latter. Plasmid pGN3 is the constitutive plasmid (CP).

The luxR* gene was first amplified using primers KW78F1 [AAC TTT ATA AGG AGG AAA AAC ATA TGA AAA ACA TAA ATG CCG AC] and KW078R [ACT **GTC GAC **TTA ATT TTT AAA GTA TGG GC]. The resulting product was then amplified using primers KW078F2 [TAT **GAA TTC **AAC TAA AGA TTA ACT TTA TAA GGA GGA AAA ACA ] and KW078R to introduce the EcoRI cut site (shown in boldface). This product was then digested with EcoRI and SalI and sub-cloned into the EcoRI and SalI cut-sites of pPROTetE-Kan-p15A. KW079F [ATA **GGT CTC **TGT GCA AAT GAA ACT CAA TAC AAC] and KW079R [ATA **GGT CTC **TGC ACA TTG GTT AAA TGG AAA GTG A] were then used to perform a whole plasmid PCR on the above to introduce a mutation GCG→GTG (Ala221→Val) in *luxR*. The resulting PCR product was then digested and blunt end ligated with BsaI to obtain pGN3. The plasmid pGN3 thus consisted of the p15A origin of replication and the ampicillin resistance marker (Ap^*r*^) (Figure 2). It consists of a copy of the luxR* gene under the control of the promoter *p*_*LtetO*-1_. In the presence of the repressor TetR, this promoter is tightly repressed, and can be induced by the addition of anhydrotetracycline (aTc). However, the *ML15A *host strain used in this experiment does not produce

TetR, and thus the luxR* gene is expressed constitutively in the host. The pGN3 plasmid was used as the constitutive plasmid (CP).

#### Feedback plasmid

pGN29 consists of a plasmid harboring the ColE1 origin of replication, chloramphenicol resistance (Cm^*r*^), a green fluorescent protein (*gfp*) and a copy of luxR* under the control of the lux promoter (*p*_*lux*_). The *gfp *gene was replaced by the *bd *oxidase gene producing the plasmid pKB1. The *bd *oxidase gene was extracted from the pTY1 plasmid by PCR using the forward primer 5'(ATA **GAA TTC **GCG ATG AGC AAG GAG TCA TG ATG TTA GAT ATA GTC G)3' and the reverse primer 5'(ATT **AAG CTT **CGC TTA GTA CAG AGA GTG GGT GTT ACG TTC AAT ATC)3'. The forward primer contained an EcoRI site (shown in boldface) and the reverse primer contained HindIII site. pGN29 and the PCR product were digested by using EcoRI and HindIII. The PCR product obtained was ligated with the digested pGN29 to obtain the positive feedback plasmid pKB1. The resulting 5.5 kb plasmid was separated using gel electrophoresis.

#### No positive feedback control plasmid

Plasmid pGN23 consists of a ColE1 origin of replication, chloramphenicol resistance and *gfp *similar to pGN29. However it contains a *p*_*LtetO*-1 _promoter, which was replaced by the *p*_*lux *_promoter. In addition, it does not contain the luxR* gene. The *bd *oxidase region was extracted from pTY1 using the same procedure as above, and was similarly ligated with the pGN23 plasmid to obtain the no positive feedback control plasmid pKB2. The resulting plasmid was 4.7 kb.

All plasmids were verified using DNA sequencing. The plasmid maps for pKB1, pKB2 and pGN3 are shown in Figure 2. Table 1 shows a list of treatments and various controls along with their designations.

**Table 1:** Experimental design to test the effect of the positive feedback.

Circuits tested	Key	Remarks
pKB1+pGN3	PF+CP	Treatment
pKB2+pGN3	NPF + CP	Control to test the effect of positive feedback
pKB2	NPF	Control to test the leakiness of the *p*_*lux *_promoter
pKB1	PF	Control to test the combined effect of the leakiness of the promoter and the positive feedback
N/A	WT	Control to test wild type expression levels

### Growth media

For all experiments, cells were grown in 5 ml of LB medium (BD Bacto™Tryptone, BD Bacto™Yeast extract, BD Bacto™agar, sodium chloride) with appropriate antibiotics (Sigma Aldrich, St. Louis, MO). Depending on the plasmid's antibiotic resistance, a combination of 100 *μ*g/ml ampicillin and 34 *μ*g/ml chloramphenicol was used.

### Preparation of inoculum

The host strain with the appropriate plasmids was grown initially in 5 ml culture tubes at 30°C in Gyrotory^®^water bath shaker (Model G76, New Brunswick Scientific, Edison, NJ) overnight in LB medium with the appropriate antibiotics. 2 ml of the overnight culture was transferred to 200 ml of fresh LB medium in 500 ml shake flasks with the appropriate antibiotics and grown at 30°C for 10-12 hours, until the culture reached an optical density of about 0.5.

### Scale up and sampling

15 ml of the inoculum prepared as above was transferred to each of the twenty four 2 l flasks containing 1 l LB medium with the appropriate antibiotics. These flasks were shaken at 220 rpm at 30°C using an Innova 4330 incubator shaker (New Brunswick Scientific, Edison, NJ). Samples were taken at 4, 8, 10, 12, 16 and 20 h. At each sampling time four 2 l flasks were removed from the shaker and the cells were peletted at 11,220 × *g *for 20 min. The pellet was stored at -80°C until further use.

### Protein sample preparation

The cell pellets were removed from -80°C and suspended in 100 ml of cell lysis buffer (100 mM Tris, 15 mM ethylenediaminetetraacetic acid (EDTA), 15 mM Benzamidine, pH 8.3). Deoxyribonuclease I (Sigma Aldrich, St. Louis, MO) and protease inhibitor cocktail (P8465, Sigma Aldrich, St. Louis, MO) were also added to the above suspension. After the sample was completely suspended in the buffer, cells were broken down by passing the sample 6 times through a nitrogen decompression based cell disruptor. The sample collected after disruption was then centrifuged at 11,220 × *g *for 20 min and the pellet was discarded. The supernatant was spun down in an ultracentrifuge (L7-65, Beckman Coulter, Fullerton, CA) at 125,000 × *g *for 4 h. The pellets were collected and stored at -80°C until further processing. The membranes collected for the different time intervals were then taken out from the -80°C freezer and 0.5 g of each sample was weighed out and solublized in 30 ml of buffer (50 mM monopotassium phosphate, 25 mM potassium chloride, 5 mM ethylenediaminetetraacetic acid (EDTA), 0.75% sucrose monododecanoate w/v, pH 6.5). The suspended sample was spun down in an ultracentrifuge at 125,000 × *g *for 2 h and the supernatant was collected. The supernatant was used to find the concentration of heme *d *in each sample by obtaining the absorbance spectrum of the sample.

### Spectroscopic measurements

All of the absorbance spectra in the UV-Visible region were obtained with a UV-Visible spectrophotometer (8453, Agilent Technologies, Santa Clara, CA) using a 1-cm path-length cuvette. For each sample, absorption spectra were obtained for both air oxidized and reduced heme *d*. The sample was reduced by adding sodium dithionite in excess. Cytochrome bd oxidase has a chlorin chromophore (heme D) exhibiting a characteristic absorption maximum at 628 nm in the fully reduced state. The bd oxidase concentration was determined from the difference in the absorption spectra between dithionite-reduced and air-oxidized bd oxidase levels using the following equation [29]:(1)

In this way, one can quantify the amount of active bd oxidase in the membrane. The absorption at 607 nm is not affected by bd oxidase and serves to eliminate any artifacts due to baseline shifts in the absorbance spectrum.

## Results and Discussion

**Figure 3:**
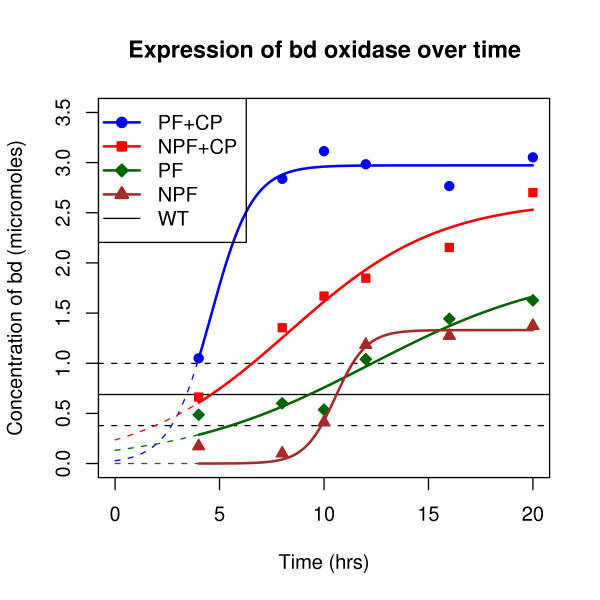
**Kinetics of bd oxidase expression**. Expression of *bd *oxidase was measured every four hours. The expression system including both the constitutive plasmid (CP) and the positive feedback plasmid (PF) showed higher expression levels and faster expression kinetics than the controls. A logistic growth curve has been fitted to the readings. For the wild type (WT), the horizontal line represents the average expression of *bd *oxidase over the experimental interval, with the dotted lines enclosing the confidence interval for the mean.

Table 2 shows the results of the experiments. Each data point corresponds to one reading of the level of bd oxidase pooled from four 1 l culture flasks. All gene circuits tested show bd oxidase levels that eventually rise above the background expression level of the wild type. It is expected that some of the cultures show bd oxidase levels lower than wild type initially, because unlike the wild type, the *ML15A *host strain is a *bd oxidase *knock out. The wild type had an average concentration of 0.68 (± 0.31) *μM *of bd oxidase as compared to 3.05 (± 0.14) *μM *for the two-plasmid systems. In addition, positive feedback has a significant effect on the kinetics of expression of *bd *oxidase. Figure 3 shows the graph of *bd *oxidase expression. In order to determine the statistical significance of the results, the following form of the logistic growth model was fitted to the data:(2)

**Table 2:** Concentration in micromoles (*μM*) of bd oxidase produced by the various gene circuits.

Time	Treatment	Controls			
(hrs)	PF+CP	NPF+CP	PF onlY	NPF only	WT
4	1.050	0.663	0.487	0.173	0.483
8	2.838	1.354	0.601	0.101	0.715
10	3.114	1.670	0.537	0.411	0.582
12	2.983	1.848	1.040	1.184	0.655
16	2.766	2.154	1.443	1.274	0.932
20	3.052	2.701	1.627	1.373	0.763

Refer Table 1 for a description of the treatment and controls.

**Figure 4:**
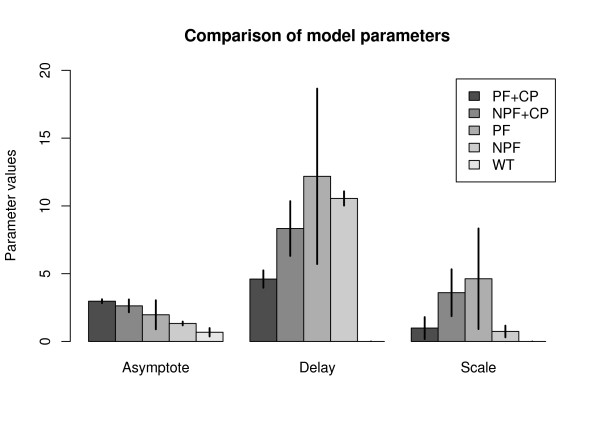
**A statistical comparison of the logistic growth curve model parameters**. The units for the Y-axis for the Asymptote (*A*) parameter are micromoles, whereas for delay (*d*) and scale (*s*) parameters have the units of hours. The error bars show two standard deviations on either side, representing the confidence intervals for the parameter values.

Here, [bd] is the concentration of bd oxidase in *μM*, *A *is the final asymptotic level of the growth curve, *d *is the time required for half-maximal expression, *s *is a scaling parameter and *t *is time in hours. This model captures all the parameters of interest of the positive feedback, including amplification (*A*), delay in expression (*d*), and system response kinetics (*s*). For the sample size of six data points per curve, the model parameters in Equation 2 were computed using a non-linear fitting function provided by the open source statistical programming language R^®^http://r-project.org. Table 3 shows the values of these parameters along with the statistics signifying the goodness-of-fit. Figure 4 shows the statistical comparison between the parameter values for the different circuits.

**Table 3:** Logistic growth model parameters for the experimental data.

Asymptote (*A*)			
**Circuit**	**Estimate**	**Std. Error**	**p-value**

PF+CP	2.9724	0.0722	2.08e-06
NPF+CP	2.6296	0.2365	0.000372
PF	1.9712	0.5349	0.0211
NPF	1.33029	0.06984	4.48e-05
WT*	0.68	0.155	2.9e-06

**Delay (*d*)**			

**Circuit**	**Estimate**	**Std. Error**	**p-value**

PF+CP	4.6043	0.3199	0.000135
NPF+CP	8.3328	1.0092	0.001174
PF	12.182	3.235	0.0197
NPF	10.55178	0.26399	2.34e-06

**Scale (*s*)**			

**Circuit**	**Estimate**	**Std. Error**	**p-value**

PF+CP	0.9892	0.4051	0.071
NPF+CP	3.5978	0.8644	0.0141
PF	4.6276	1.8552	0.0672
NPF	0.73923	0.21563	0.0266

For the WT, the average value was assumed to be the asymptotic value (*A*), while the delay and scale parameters were not estimated.

From the data and the computed model parameters (Figures 3 and 4), it is clear that positive feedback has a significant effect on the production kinetics of bd oxidase. This behavior has been previously reported by several researchers in literature for various cytosolic and indicator proteins, and we confirm that it translates to the expression of membrane proteins as well. Qualitatively, our result is slightly different from previous work because in this case, there is a steric limit on the amount of membrane protein that can be produced, governed by the amount of membrane itself. Since the asymptotic membrane protein level is naturally capped, the membrane localization kinetics appear to speed up.

Ideally, one would need replicates to determine the variability from sample to sample for each of the data points. While replicates for each time point would have led to greater confidence in the actual values of the model parameters, as a series of six time points they are sufficient to show that there exist significant statistical differences between the treatment and the various controls. Moreover, the non-availability of the time points is a result of the original problem: A large culture volume is needed to produce a sample of membrane protein. In our case, each time point is a pooled sample of four 1 l samples which leads to one measurable reading.

Nevertheless, within reason, certain conclusions can be drawn. Firstly, all circuits tested produced more bd oxidase than the wild type. This result was expected since *bd *oxidase is typically expressed in microaerobic conditions, whereas the conditions in our experiment were aerobic. Secondly, all plasmids used were medium copy plasmids, and by virtue of having multiple copies of the same gene, one can expect a production level higher than that seen in the wild type. Thirdly, the constitutive plasmid (i.e. the constitutive promoter) plays a greater role in the final level of gene expression rather than the positive feedback, since there is no significant difference between the asymptote parameter (*A*) of the treatment (PF+CP) and the NPF+CP control, while there is a significant difference between the NPF+CP and the NPF controls. Finally, the *p*_*lux *_promoter, which is known to be leaky can cause undesirable expression of the membrane protein, which can lead to high expression levels if there is a positive feedback available to amplify this leaky expression. This is an important consideration when expressing toxic proteins. The positive feedback really shows its benefits in reducing the delay in producing the protein. In other words, the positive feedback can reduce the time required to saturate a membrane with membrane protein, when viewed in comparison with the NPF+CP control. However, the positive feedback might not be sufficient by itself and requires a driver plasmid for maximum productivity.

The scaling parameter (*s*) gives an idea of the rate at which a positive feedback can enable a circuit to switch from the off state to the on state. As the scaling parameter tends to zero, the switching behavior looks more 'digital', i.e. with instantaneous switching between states. In this case, the parameter values are difficult to interpret. The treatment seems to have a high switching speed (low scaling parameter value), but at the same time the NPF control also seems to have a similar behavior, which could be a computational artifact due to the low final expression level of *bd *oxidase in this case.

## Conclusions

One of the significant outcomes of the positive feedback expression system is that the time required to reach steady state levels of bd oxidase membrane protein reduces from 20 h (in the no feedback control case) to about 8 h. There are, of course, several other factors that determine the precise numerical values of this performance gain, including promoter strength, plasmid copy number and the specific strain used. While these experiments do not have sufficient precision to quantify these performance gains, they do show that the positive feedback-based system accrues statistically significant gains.

One of the goals of synthetic biology is to develop reusable gene regulatory systems. The positive feedback-based system we have developed is modular and can be readily extended as a building block in other protein expression systems. It can also be used to enhance existing protein expression systems, which use inducible promoters or exogenous polymerases such as T7 RNAP. It is therefore an attractive alternative to increase the yields of difficult-to-produce proteins such as membrane proteins.

## Competing interests

The authors declare that they have no competing interests.

## Authors' contributions

KDB and RBG conceived the experiments. KB did the experiments and wrote the manuscript draft. GJN helped with plasmid construction and with manuscript revision. KY helped with the experimental protocols. KB and KDB analyzed the data. All authors read and approved the final manuscript.

## References

[B1] Lee SK Chou H Ham TS Lee TS Keasling JD Metabolic engineering of microorganisms for biofuels production: from bugs to synthetic biology to fuels Curr Opin Biotechnol 2008 19 6 556 63 10.1016/j.copbio.2008.10.014 18996194

[B2] Ro DK Paradise EM Ouellet M Fisher KJ Newman KL Ndungu JM Ho KA Eachus RA Ham TS Kirby J Chang MCY Withers ST Shiba Y Sarpong R Keasling JD Production of the antimalarial drug precursor artemisinic acid in engineered yeast Nature 2006 440 7086 940 943 10.1038/nature04640 16612385

[B3] Levskaya A Chevalier AA Tabor JJ Simpson ZB Lavery LA Levy M Davidson EA Scouras A Ellington AD Marcotte EM Voigt CA Synthetic biology: engineering Escherichia coli to see light Nature 2005 438 7067 441 442 10.1038/nature04405 16306980

[B4] Tecon R Wells M Meer JR van der A new green fluorescent protein-based bacterial biosensor for analysing phenanthrene fluxes Environ Microbiol 2006 8 4 697 708 10.1111/j.1462-2920.2005.00948.x 16584481

[B5] Balagadde FK Song H Ozaki J Collins CH Barnet M Arnold FH Quake SR You L A synthetic Escherichia coli predator-prey ecosystem Mol Syst Biol 2008 4 187 10.1038/msb.2008.24 PMC2387235 18414488

[B6] Cox RS Surette MG Elowitz MB Programming gene expression with combinatorial promoters Mol Systems Biol 2007 3 145 10.1038/msb4100187 PMC2132448 18004278

[B7] Alper H Fischer C Nevoigt E Stephanopoulos G Tuning genetic control through promoter engineering Proc Natl Acad Sci USA 2005 102 36 12678 83 10.1073/pnas.0504604102 PMC1200280 16123130

[B8] Gardner TS Cantor CR Collins JJ Construction of a genetic toggle switch in Escherichia coli Nature 2000 403 6767 339 342 10.1038/35002131 10659857

[B9] Basu S Mehreja R Thiberge S Chen MT Weiss R Spatiotemporal control of gene expression with pulse-generating networks Proc Natl Acad Sci USA 2004 101 17 6355 6360 10.1073/pnas.0307571101 PMC404049 15096621

[B10] Elowitz MB Leibler S A synthetic oscillatory network of transcriptional regulators Nature 2000 403 6767 335 338 10.1038/35002125 10659856

[B11] Win MN Smolke CD Higher-order cellular information processing with synthetic RNA devices Science 2008 322 5900 456 460 10.1126/science.1160311 PMC2805114 18927397

[B12] Benner SA Sismour AM Synthetic biology Nat Rev Genet 2005 6 7 533 543 10.1038/nrg1637 PMC7097405 15995697

[B13] Alon U Biological networks: the tinkerer as an engineer Science 2003 301 5641 1866 1867 10.1126/science.1089072 14512615

[B14] Andrianantoandro E Basu S Karig DK Weiss R Synthetic biology: new engineering rules for an emerging discipline Mol Syst Biol 2006 2 20060028. 10.1038/msb4100073 PMC1681505 16738572

[B15] Bhalerao K Synthetic gene networks: The next wave in biotechnology? Trends Biotechnol 2009 27 6 368 74 10.1016/j.tibtech.2009.03.003 19409633

[B16] Maeda YT Sano M Regulatory dynamics of synthetic gene networks with positive feedback J of Mol Biol 2006 359 4 1107 1124 10.1016/j.jmb.2006.03.064 16701695

[B17] Becskei A Seraphin B Serrano L Positive feedback in eukaryotic gene networks: cell differentiation by graded to binary response conversion The EMBO journal 2001 20 10 2528 2535 10.1093/emboj/20.10.2528 PMC125456 11350942

[B18] Williams JW Cui X Levchenko A Stevens AM Robust and sensitive control of a quorum-sensing circuit by two interlocked feedback loops Mol Syst Biol 2008 4 234 10.1038/msb.2008.70 PMC2615304 19096361

[B19] Nistala GJ Wu K Rao CV Bhalerao KD A modular positive feedback-based gene amplifier J Biol Eng 2010 4 4 10.1186/1754-1611-4-4 PMC2845093 20187959

[B20] Wallin E von Heijne G Genome-wide analysis of integral membrane proteins from eubacterial, archaean, and eukaryotic organisms Protein Sci 1998 7 4 1029 1038 10.1002/pro.5560070420 PMC2143985 9568909

[B21] Klabunde T Hessler G Drug design strategies for targeting G-protein-coupled receptors Chembiochem 2002 3 10 928 944 10.1002/1439-7633(20021004)3:10<928::AID-CBIC928>3.0.CO;2-5 12362358

[B22] Lundstrom K Structural genomics on membrane proteins: mini review Comb Chem High Throughput Screen 2004 7 5 431 439 10.2174/1386207043328634 15320710

[B23] Wagner S Klepsch MM Schlegel S Appel A Draheim R Tarry M Högbom M van Wijk KJ Slotboom DJ Persson JO de Gier JW Tuning Escherichia coli for membrane protein overexpression Proc Natl Acad Sci USA 2008 105 38 14371 6 10.1073/pnas.0804090105 PMC2567230 18796603

[B24] Rice CW Hempfling WP Oxygen-limited continuous culture and respiratory energy conservation in Escherichia coli J Bacteriol 1978 134 115 124 10.1128/jb.134.1.115-124.1978 PMC222225 25879

[B25] Minghetti KC Gennis RB The two terminal oxidases of the aerobic respiratory chain of Escherichia coli each yield water and not peroxide as a final product Biochem Biophys Res Commun 1988 155 243 248 10.1016/s0006-291x(88)81075-1 2843179

[B26] Lorence RM Gennis RB Spectroscopic and quantitative analysis of the oxygenated and peroxy states of the purified cytochrome d complex of Escherichia coli J Biol Chem 1989 264 13 7135 7140 2540176

[B27] Poellinger KA Lee JP JVP Jr Greenberg EP Intragenic suppression of a luxR mutation: characterization of an autoinducer-independent LuxR FEMS microbiology letters 1995 129 97 101 10.1016/0378-1097(95)00145-U 7781994

[B28] Lutz R Bujard H Independent and tight regulation of transcriptional units in Escherichia coli via the LacR/O, the TetR/O and AraC/I1-I2 regulatory elements Nucleic acids research 1997 15;25 6 1203 10 10.1093/nar/25.6.1203 PMC146584 9092630

[B29] Borisov V Arutyunyan AM Osborne JP Gennis RB Konstantinov AA Magnetic circular dichroism used to examine the interaction of Escherichia coli cytochrome bd with ligands Biochemistry 1999 38 2 740 750 10.1021/bi981908t 9888814

